# Improvement of the Trivalent Inactivated Flu Vaccine Using PapMV Nanoparticles

**DOI:** 10.1371/journal.pone.0021522

**Published:** 2011-06-29

**Authors:** Christian Savard, Annie Guérin, Karine Drouin, Marilène Bolduc, Marie-Eve Laliberté-Gagné, Marie-Christine Dumas, Nathalie Majeau, Denis Leclerc

**Affiliations:** Department of Microbiology Infectiology and Immunology, Infectious Disease Research Centre, Laval University, Quebec City, Canada; Virginia Polytechnic Institute and State University, United States of America

## Abstract

Commercial seasonal flu vaccines induce production of antibodies directed mostly towards hemaglutinin (HA). Because HA changes rapidly in the circulating virus, the protection remains partial. Several conserved viral proteins, e.g., nucleocapsid (NP) and matrix proteins (M1), are present in the vaccine, but are not immunogenic. To improve the protection provided by these vaccines, we used nanoparticles made of the coat protein of a plant virus (papaya mosaic virus; PapMV) as an adjuvant. Immunization of mice and ferrets with the adjuvanted formulation increased the magnitude and breadth of the humoral response to NP and to highly conserved regions of HA. They also triggered a cellular mediated immune response to NP and M1, and long-lasting protection in animals challenged with a heterosubtypic influenza strain (WSN/33). Thus, seasonal flu vaccine adjuvanted with PapMV nanoparticles can induce universal protection to influenza, which is a major advancement when facing a pandemic.

## Introduction

Influenza epidemics cause between 250,000 and 500,000 deaths every year worldwide (http://www.who.int/en/). Many of these deaths could be prevented by vaccination programs but vaccine producers are currently unable to keep up with increasing demand and the need for new vaccines. Most seasonal vaccines against influenza are based on trivalent inactivated vaccine (TIV), which induces an antibody response towards the highly variable surface glycoproteins hemagglutin (HA) and neuraminidase (NA) [1]. The effectiveness of neutralizing antibody generated by these vaccines declines over time as circulating viruses accumulate mutations in response to immune pressure [2]. As a consequence, the immunity engendered by TIV is partial, as it offers no protection against antibody-escaped variants or new pandemic influenza A viruses originating from non-human reservoir [3], [4], [5]. Other issues that limit the utility of TIV include the lack of a reliable method to estimate future influenza evolution, and the long lead time between the selection of vaccine strains and release of vaccine onto the market, which could help explain the high degree of mismatch between circulating and vaccine strains. Current vaccine production is also constrained by the absolute requirement for specialized egg-based production facilities [6].

Recent human infection by porcine A (H1N1) subtype and the highly pathogenic avian A (H5N1) influenza virus has led to renewed interest in the development of universal vaccines that would confer heterosubtypic immunity regardless of subtype or strain. Such cross-protective immunity is induced to some extent during natural infection, and is mediated mainly by CD8+ cytotoxic T lymphocytes (CTL), which recognize conserved internal components of the virus and cross reactive antibody [7]–[12]. This immune response can promote early virus elimination and decrease morbidity [13]. Unfortunately, commonly used inactivated vaccines do not induce CTL efficiently. Addition of adjuvant is one possible option to enhance the immunogenicity and efficiency of conventional vaccines [14].

In this study, we evaluated the capacity of nanoparticles made of papaya mosaic virus (PapMV) coat protein (CP) to act as an adjuvant for TIV. The expression of PapMV CP in bacteria leads to self-assembly and formation of virus-like particles (VLP, or nanoparticles) composed of several hundred recombinant CP subunits organized in a repetitive and ordered manner [15]. PapMV Nanoparticles can be used as an epitope display system that is very immunogenic, even in the absence of external adjuvant and lead to production of antibodies directed towards the surface-exposed peptide, thus providing protection [16], [17]. Engineered PapMV nanoparticles with CTL epitopes induce efficient cross presentation of the epitope on MHC class I [18], and induce protection against viral infection [19]. Based on this strong immunogenicity of PapMV nanoparticles, we evaluated their specific capacity to improve TIV efficacy, as well as their potential use as an adjuvant for seasonal influenza vaccines.

## Results

### PapMV nanoparticles are recognized by immune cells and transported to lymph nodes

**Figure 1:**
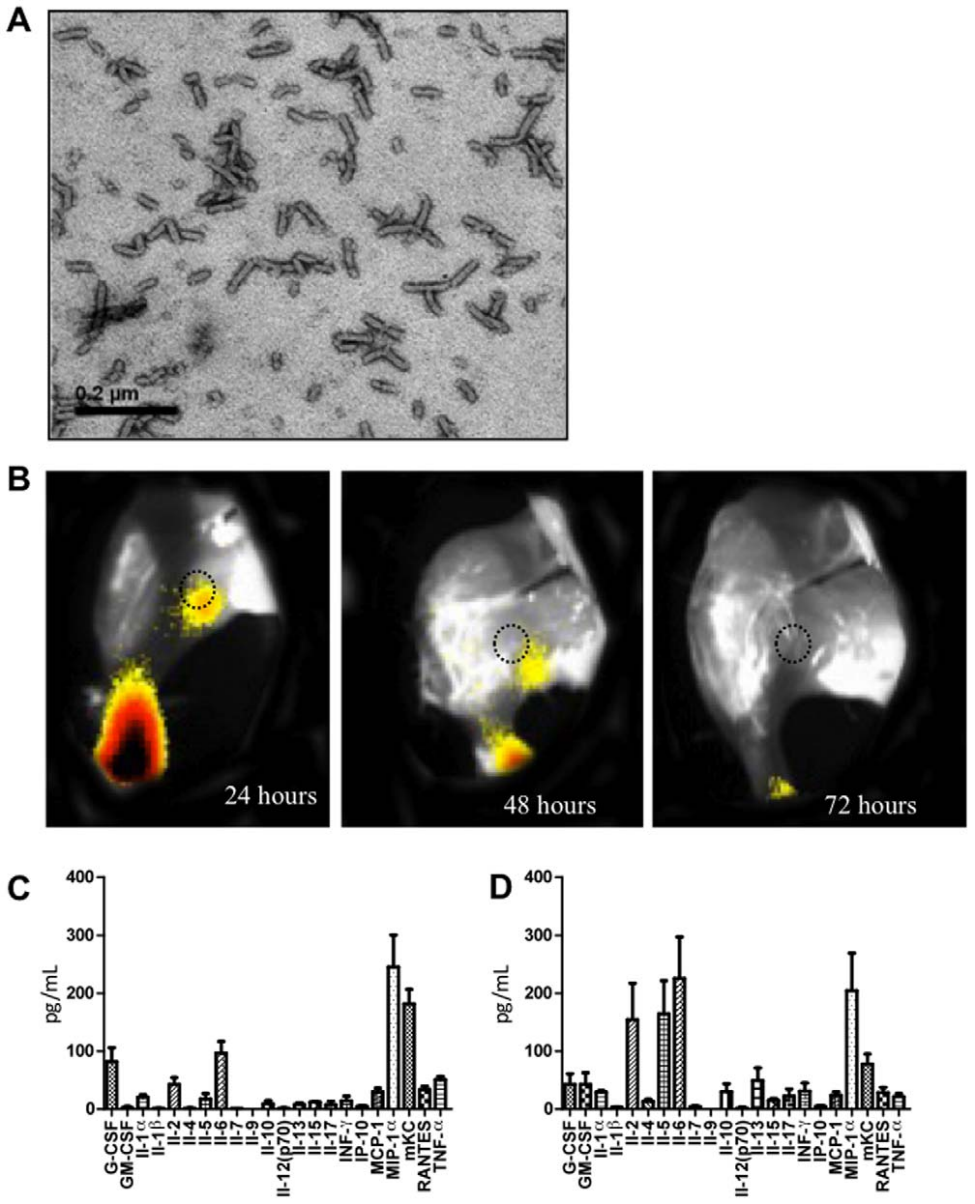
PapMV nanoparticles and the secretion of T_H1_/T_H2_ cytokines. **A,** Observation of adjuvant PapMV nanoparticles by electron microscopy. *Bar* 0.2 µm. **B,** In vivo imaging of fluorescently labeled PapMV nanoparticles. Data are presented as pseudocolor images indicating fluorescence (Alexa@680) intensity, with a gradation from red (more intense) to yellow, superimposed over gray-scale reference photographs of the left inferior member of the treated mouse. Images were taken at 24, 48 and 72 h post-injection. The proximal popliteal lymph node is indicated with a dotted circle. At 24 h, a strong signal is detected in the foot pad of the animal where the fluorescent protein was injected. **C,D** Cytokine/chemokine profile of splenocytes reactivated with PapMV nanoparticles (100 µg/ml) isolated after one (**C**) or two (**D**) subcutaneous immunizations.

We produced PapMV nanoparticles comprised of PapMV CP using the bacterial expression vector pET-3D (Novagen) as described previously [15]–[17]. SDS-PAGE confirmed production of a single, homogenous protein of 30 kDa (Figure S1A) that self-assembled into nanoparticles (Figure 1A) with an average length of 70 nm as measured by dynamic light scattering (DLS) (Figure S1B). We previously reported that PapMV nanoparticles, alone or fused to a peptide, are immunogenic [16], [17] and are taken up by dendritic cells [19]. To illustrate the speed of capture of PapMV nanoparticles by immune cells, we injected labeled nanoparticles into the footpad of Balb/C mice. The proximal popliteal lymph node became fluorescent 24 hours after injection (Figure 1B). The signal declined progressively over the subsequent 48 hours, suggesting that the nanoparticles are rapidly degraded. We also evaluated the cytokine/chemokine profile secreted by spleen cells following one or two subcutaneous injections into the back of the neck of these animals. Reactivation of spleen cells of mice immunized once led to the secretion of MIP-1a and mKC (Figure 1C). We measured lower but significant amounts of IL-6, G-CSF, TNF-α, IL-2, RANTES, MCP-1, IL-1α, Il-5, IFN-γ and IL-17 in these mice. Two immunizations also led to an increase in MIP-1a and mKC levels, as well as abundant secretion of IL-2, 5 and 6 (Figure 1D). Lower but significant levels of IL-13, G-CSF, GM-CSF, IFN-γ, Il-10, IL-1α, RANTES, MCP-1, IL-17, TNF-α and Il-4 were also detected. This result suggests that PapMV nanoparticles are perceived efficiently by the immune system and trigger secretion of a balanced T_H1_/T_H2_ cytokine profile. PapMV VLP could thus be considered as a pathogen-associated molecular pattern (PAMP) that can be potentially used as an adjuvant for improvement of TIV.

### Improvement of TIV humoral response using PapMV nanoparticles

**Figure 2:**
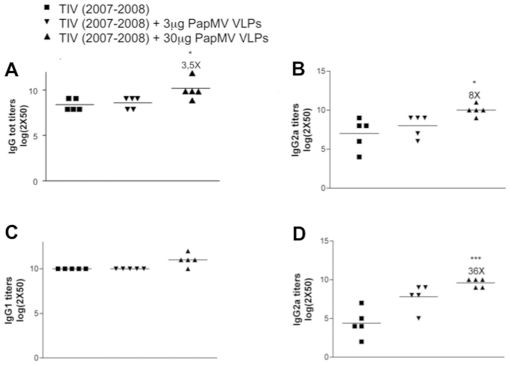
PapMV nanoparticles improve the humoral response of TIV (2007–2008). Balb/C mice (5 per group) were immunized once with 1/5 of the human dose of trivalent inactivated vaccine (TIV) (2007–2008) alone or with PapMV nanoparticles (3 or 30 µg). IgG titers were evaluated by ELISA 14 days after immunization. **a**–**c** Response to TIV (2007–2008): **A,** Total IgG, **B,** IgG2a and **C,** IgG1. **D,** IgG2a response to a recombinant GST-NP (A/WSN/33-H1N1). * p< 0.05, ** p< 0.01 and *** p< 0.001.

To test our hypothesis, we set up an immunization program with TIV (2007–2008) alone or adjuvanted with either 3 or 30 µg of PapMV nanoparticles. Balb/c mice (5/group) were immunized twice by the subcutaneous route with a 2-week interval. The humoral response against TIV and purified recombinant GST-NP (Figure 2) was measured by ELISA. The recombinant GST-NP antigen used for the ELISA is derived from the influenza strain WSN/33 (Figure S2).

The addition of 30 µg PapMV nanoparticles was more efficient than 3 µg in improving the humoral response to TIV (2007–2008); we measured a 3.5-fold increase in the amount of total IgG (Figure 2A), and a 8-fold increase in IgG2a isotype directed towards TIV (2007–2008) antigen (Figure 2B). Interestingly, the amount of isotype IgG1 was not significantly improved by the presence of PapMV nanoparticles (Figure 2C).

TIV (2007–2008) is composed of split influenza virus that contains the structural protein NP. The NP component of TIV is not very immunogenic but the addition of the PapMV nanoparticles increased the immune response directed to this highly conserved influenza antigen by 36 fold (Figure 2D), thus showing that PapMV nanoparticles improve the T_H1_ immune response directed towards the conserved influenza structural protein NP. These results suggest that, in contrast to alum, which is unable to improve the immune response of TIV (Figure S3), PapMV nanoparticles induce a T_H1_ response to TIV.

We repeated this experiment using a similar immunization protocol using TIV (2008–2009) and TIV (2009–2010), which contains different strains of influenza, to show that PapMV nanoparticles can act as an effective adjuvant for any TIV. As expected, we observed a significant increase in total IgG (>3x), and IgG2a (>4x) directed towards TIV (2008–2009) (Figure S4A–B). PapMV nanoparticles also improved significantly IgG2a directed to the conserved protein NP (>16x) (Figure S4C). With TIV (2009–2010), we showed improvements in total IgG (>8x) (Figure S5A) and IgG2a (16x) (Figure S5B) directed to TIV (2009–2010), as well as total IgG titers directed towards the pandemic influenza vaccine 2009 (Figure S5D). Furthermore, IgG2a directed towards GST-NP were detected only in the adjuvanted group (Figure S5C).

**Figure 3:**
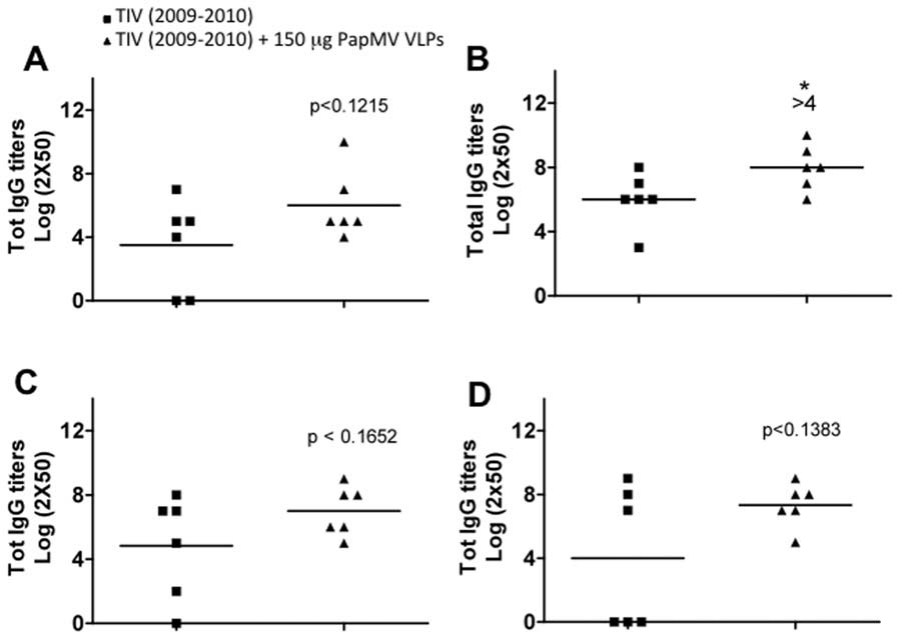
PapMV nanoparticles improve the humoral response of TIV (2009–2010) in ferrets. Male ferrets (6 per group) were immunized twice with a 3-week interval with a human dose of TIV (2009–2010) alone or with PapMV nanoparticles (150 µg). Serum IgG titers were evaluated by ELISA after 21 days. **A,** total IgG to TIV (2009–2010) after one immunization; **B,** total IgG to TIV (2009–2010) after two immunizations; **C,** total IgG to TIV (2007–2008); **D,** total IgG to a recombinant GST-NP (A/WSN/33-H1N1).

To confirm this result in another animal model, we immunized ferrets (6 per group) twice with a 3-week interval with one human dose of TIV (2009–2010) alone or adjuvanted with 150 µg of PapMV nanoparticles. We showed that PapMV nanoparticles improved the total IgG titers to TIV (2009–2010) already after one immunization (Figure 3A), reaching a significant 4-fold increase after one booster (Figure 3B). The ELISA against TIV (2008–2009), which contains related but distinct strains of influenza, using the same serum showed a tendency towards improvement in the presence of the adjuvant (p< 0.1652) (Figure 3C). Similarly, we notice a tendency towards improvement of the IgG response to GST-NP (p< 0.1383) (Figure 3D), which is consistent with the results obtained in mice. The lack of significant difference in the average of the two last groups is probably related to the small number of animals per group used in the experiment. Interestingly, we noticed a significant difference in the variance between the non adjuvanted and the adjuvanted group in the amount of IgG directed to the GST-NP protein (Figure 3D).

**Figure 4:**
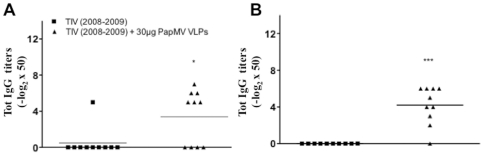
PapMV nanoparticles increase the breadth of the humoral response directed towards TIV (2008–2009). Balb/C mice (10 per group) were immunized once with 1/5 of the human dose of TIV (2007–2008) alone or with 30 µg PapMV nanoparticles. **A,** Total IgG directed towards intact virion of influenza strain WSN/33. **B,** Total IgG directed to intact virion of the strain A/Kentucky/91 (H3N8). * p< 0.05 and *** p< 0.001.

To demonstrate the capacity of PapMV nanoparticles to improve the humoral response to conserved epitopes located on the surface glycoproteins of influenza, we performed ELISA with the serum generated from the immunization protocol with TIV (2008–2009) and 30 µg PapMV nanoparticles toward the heterosubtypic mouse-adapted strain WSN/33 (H1N1) or the horse strain A/Kentucky/91 (H3N8). Intact virus was used to coat the ELISA plate, restricting the epitopes available for binding antibodies to the HA and NA proteins located at the surface of the virus. Interestingly, 6 out of 10 mice in the adjuvanted groups reacted to the WSN/33 coating (Figure 4A), and 9 out of 10 sera reacted to the horse A/Kentucky/91 (H3N8) strain (Figure 4B). A similar observation was made with the serum of mice vaccinated with adjuvanted TIV (2009–2010), with the amount of IgG able to cross react with the pandemic H1N1 Flu vaccine being increased 32 fold (Figure S5D). The cross reactivity of the serum of the adjuvanted mice suggests that PapMV nanoparticles are able to increase the breadth of the humoral response to include epitopes that are common to heterosubtypic strains that are unrelated to the strain present in the vaccine.

**Figure 5:**
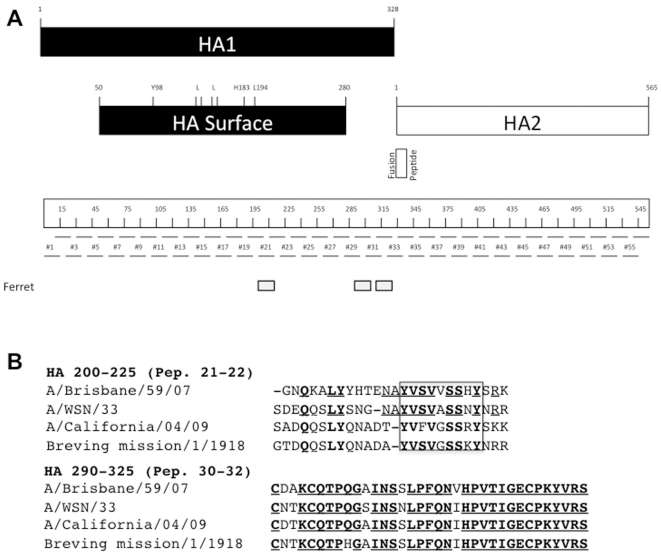
Summary of the immunoblot analysis using serum of ferrets immunized with TIV alone or with PapMV nanoparticles. **A,** Schematic representation of HA protein sequence and position of the different 15-aa peptides on the HA sequence. Small gray rectangles represent peptides that reacted exclusively with serum of ferrets immunized with TIV (2009–2010) + 150 µg PapMV nanoparticles, or peptides that reacted only with serum of mice immunized with TIV (2008–2009) + 30 µg PapMV nanoparticles. Black rectangles represent the extent of peptides that reacted in both species. **B,** Amino acid sequence alignment of peptidic regions that reacted with serum of both species. A/Brisbane/59/07 is the H1N1 vaccine strain, A/WSN/33 is the mouse-adapted strain, A/California/04/09 is the 2009 pandemic strain, and A/Brevig Mission/1/1918 is the Spanish flu strain. Identical amino acids are in bold and underlined. A conserved region of HA is boxed.

To confirm that use of the adjuvant increases the breadth of the humoral response to conserved epitopes of HA, we performed an immunoblot on 56 peptides of 15 amino acids in length overlapping each other by 5 amino acids, and covering the entire amino acid sequence of WSN/33 HA. The peptides were spotted onto a glass plate and hybridized with serum of ferrets immunized with TIV alone or adjuvanted with PapMV nanoparticles. A very weak signal on a few peptides was obtained when the peptides were hybridized with pre-serum or with serum of ferrets immunized with TIV (2009–2010)(Figure S6). When using serum of animals vaccinated with adjuvanted vaccine, we considered positive signals to be those at least 3 fold higher than the background obtained with pre-serum or serum obtained from animals vaccinated with TIV (2009–2010) alone. We found that three additional peptides were recognized only by the adjuvanted ferret serum (Figure 5A). The location of these three regions corresponds to HA 200–225 and HA 290–325—conserved regions that are common to the H1N1 strain Brisbane/59/07 found in TIV (2009–2010) and the WSN/33 mouse-adapted strain (Figure 5B). These same regions are also common to the H1N1 pandemic strain of 2009 and the H1N1 pandemic strain of 1918. Interestingly, HA 290–325 is located near the signal peptide sequence of HA—a region known to be a good target for the development of a universal influenza vaccine [20].

### PapMV nanoparticles increase secretion of IFN-γ by T lymphocytes

**Figure 6:**
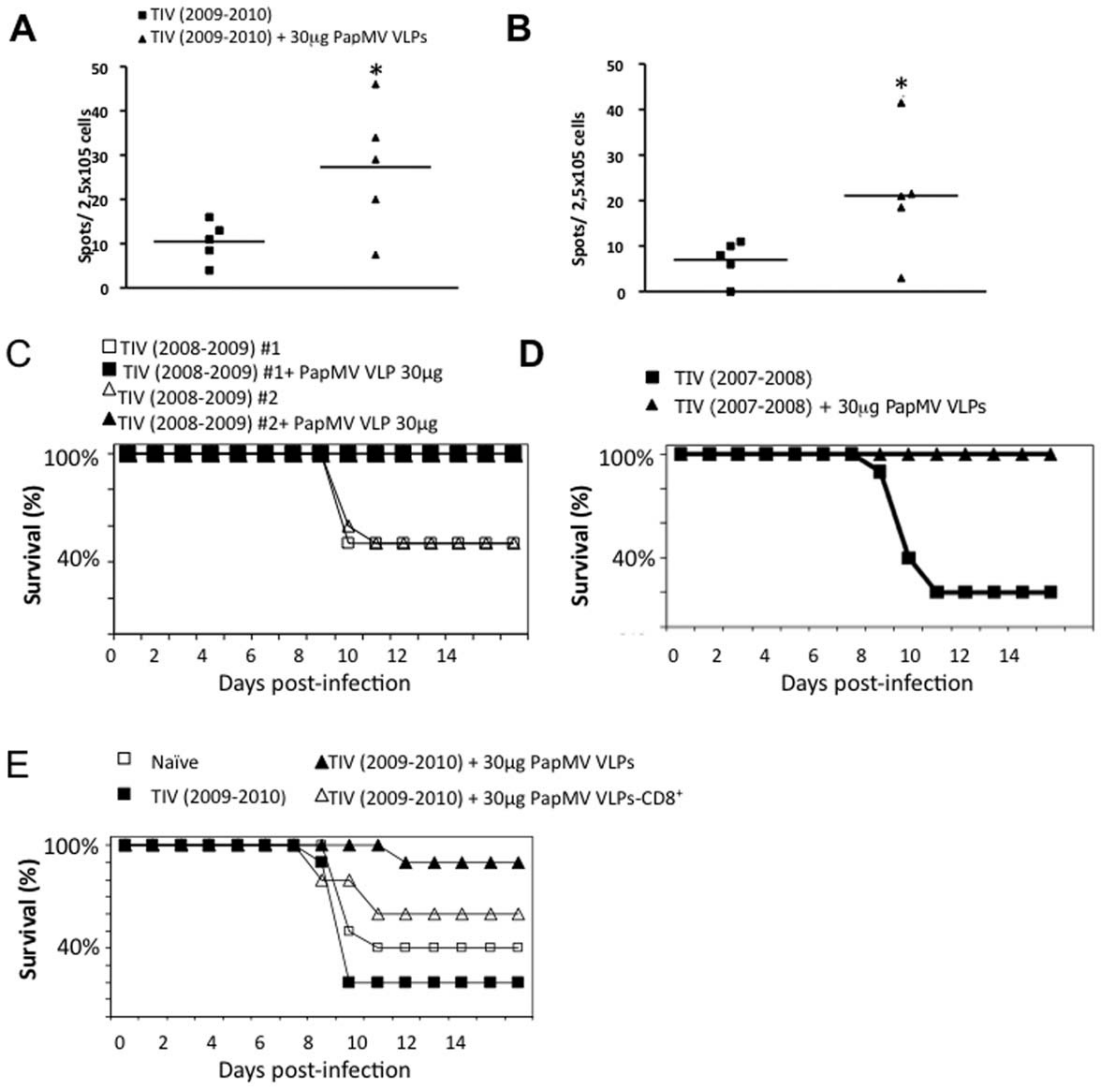
PapMV nanoparticles trigger a CTL response to conserved influenza epitopes. Balb/C mice (5 per group) were immunized twice with a 14-day interval with 1/5 of the human dose of TIV (2009–2010) alone or with 30 µg of PapMV nanoparticles. Spleens were collected 14 days after the boost. We used purified recombinant GST-NP (**A**) or GST-M1 protein derived from the WSN/33 strain (**B**) to perform an ELISPOT assay. * P <0.05. (**C**) PapMV nanoparticles improve survival to a sub-lethal challenge with a heterosubtypic strain. Balb/C mice (10 per group) were immunized with TIV (2008–2009) from two different companies (#1 or 2) alone or with 30 µg of PapMV nanoparticles. Mice were challenged with 1LD50 of A(H1N1)/WSN/33 influenza virus, 2 weeks after the final boost and were followed for a 14-day period. (**D**) A similar protocol was also followed with mice immunized once with TIV (2007–2008) alone or adjuvanted with 30 µg PapMV nanoparticles except that the infection with the heterosubtypic strain WSN/33 was performed 10 months after the immunization. (**E**) A immunization protocol similar to that described in **c** was performed with TIV (2008–2009) alone or adjuvanted with 30 µg of PapMV nanoparticles. The depletion of CD8+ cells was performed at days -3 and -1 before the challenge and 14 days after the boost.

The induction of high levels of IgG2a to conserved antigens of TIV by PapMV nanoparticles suggests that the adjuvant induces a T_H1_ response. To verify the efficacy of the adjuvant to improve secretion of IFN-γ—a good T_H1_ marker—we performed an ELISPOT assay using the highly conserved influenza proteins M1 and NP to reactivate T lymphocytes. As before, Balb/c mice (5/group), were vaccinated with TIV (2008–2009) alone or with PapMV nanoparticles. Splenocytes were collected 14 days after the boost. The ELISPOT assay showed a significant increase in the number of T cells secreting IFN-γ in the adjuvanted groups when reactivated with GST-NP (Figure 6A) or GST-M1 (Figure 6B), which supports the hypothesis that PapMV nanoparticles trigger a CTL response.

### PapMV nanoparticle adjuvant induces protection from infection with a heterosubtypic strain

The immunological characterization presented above shows that using PapMV nanoparticles as an adjuvant can trigger a T_H1_ response to highly conserved proteins of influenza (e.g., NP and M1) that extends the humoral response to conserved regions of the HA protein found on heterosubtypic strains of the virus. It is therefore expected that PapMV nanoparticles will improve the protection afforded TIV from infection with a heterosubtypic strain of influenza. To test this hypothesis, we immunized mice twice with TIV (2008–2009) from two different commercial sources, alone or adjuvanted with 30 µg of PapMV nanoparticles. The mice were then challenged with the mouse-adapted strain WSN/33. We found that mice treated with the adjuvanted vaccine did not lose weight (Figure S7A), showed no viral symptoms (Figure S7B) and were afforded 100% protection (Figure 6C) compared to mice vaccinated with TIV alone, which were strongly affected by infection with WSN/33.

To see if a memory response was induced by the adjuvant, we immunized mice once with TIV (2007–2008) alone or with TIV adjuvanted with 30 µg of PapMV nanoparticles and performed the challenge 10 months after immunization. The improvement in the immune response in the adjuvanted groups was maintained even 10 months after the immunization (Figure S8A–C). Upon challenge with the WSN/33 strain, we observed no weight loss (see Figure S8D) or symptoms (Figure S8E), and 100% of the animals immunized with the adjuvanted vaccine (Figure 6D) were protected, in contrast with mice vaccinated with TIV alone, which showed only 20% survival (Figure 6D).

Finally, to monitor the importance of the CD8+ mediated immune response to protection induced by PapMV nanoparticles adjuvant, we immunized mice (10/group) with TIV (2008–2009) alone, 2 groups with TIV (2008–2009) adjuvanted with 30 µg of PapMV nanoparticles and one control group with saline. One of the adjuvanted groups was further treated at days -3 and -1 before infection with WSN/33 with a monoclonal antibody directed towards CD8 to deplete CTL. Infection of the immunized mice with WSN/33 revealed that CTL depletion affected the protection observed in the adjuvanted groups partially but significantly (Figure 6E). Depletion of CD8+ had a minor effect on weight loss (Figure S9A) but enhanced the symptoms induced by the infection (Figure S9B). An imperfect but significant correlation between the levels of IgG directed to WSN/33 virus and protection was observed, suggesting that antibodies might play an effective role in protecting mice from the challenge (Figure S9C).

## Discussion

The efficiency of TIV immunization decreases as the mismatch between the vaccine strain and the circulating strain increases [3]–[5]. The use of adjuvants in vaccines is an attractive approach to increasing the cross-reactivity of influenza vaccines [21). It is widely accepted that protection against seasonal influenza correlates highly with the level of serum antibodies, which are directed mainly against HA protein. Using PapMV nanoparticles as an adjuvant, we increased the global humoral response through the increase of total IgG titers, and particularly the IgG2a subclass against TIV. IgG2a is more effective in preventing intracellular virus replication since it is more efficient in complement activation and antibody-dependent cellular immunity [22], [23]. This different activation capacity can be explained by the stronger affinity of IgG2a for the complement [24], [25] and Fc receptor [26], [27] than the IgG1 subclass. For the reasons stated above, T_H1_-type and IgG2a-dominated humoral responses are preferred over T_H2_-type responses for protection against influenza infection [26], [28]. Other adjuvants have been shown to increase the antibody response to flu vaccines, but many of them, like MF-59, have a bias towards T_H2_ rather than the T_H1_-like response promoted by PapMV nanoparticles [21]. This data is consistent with the profile of cytokines and chemokines that were induced in splenocytes stimulated with PapMV nanoparticles, where abundant pro-inflammatory cytokines (MIP-1a and KC), T_H1_ (IL-2) and TH2 cytokines (IL-5, IL-6) were induced.

Our immunoblot analysis demonstrated that PapMV nanoparticles increased the breadth of the antibody response to HA as previously shown for MF-59 [29]. A recent study on HIV antigens adsorbed on particulate adjuvant showed that surface rearrangement of the target protein leads to unmasking of cryptic epitopes, which is important for protection against heterosubtypic strain [30]. The same phenomenon was observed using PapMV nanoparticles as an adjuvant in mice and ferrets. Antibodies directed towards a highly conserved pocket in the stem region of HA containing the fusion peptide were revealed using PapMV nanoparticles. Such an antibody has been shown to block infection by inserting its heavy chain into this region, and this could potentially interfere with membrane fusion rather than cell attachment [20]. Recent studies with monoclonal antibodies have shown that a domain in the stalk region of HA is conserved across a number of subtypes [31], [32] and is protective against lethal challenge of H5N1 and H1N1 in mice. The improvement in the antibody response with PapMV nanoparticles correlates with the protection against a heterosubtypic strain, which can also be attributed to a cross reaction with conserved epitopes on the surface of infected cells that could be useful in the control of infection via an antibody-mediated cellular response [33], [34].

The use of PapMV nanoparticles also increased the IFN-γ mediated immune response against highly conserved influenza proteins within different subtypes, such as NP and M1 [35], [36]. Also, it has been shown previously that antibodies directed towards an internal protein of the virus particle can contribute to protection from influenza challenge via the involvement of complement and antibody dependent cellular mechanisms [37]–[40]. In this study, we measured increased protection against a distant heterosubtypic strain (WSN/33) resulting from the use of the adjuvant. Protection was still efficient even 10 months after immunization. Heterosubtypic protection is mediated by both CD4+ and CD8+ T cells, and is directed mostly against internal viral proteins, although the CD8+ subset is generally considered to be more important [41], [42]. In the present study, results from CD8+ cell depletion experiments suggest that CD8+ T cells play an important role in the enhanced protection observed.

One of the main concerns surrounding the use of adjuvants is the potential toxicity of these molecules, which is usually related to the development of local reactions due to the induction of a strong inflammatory response [43]. In most cases, inflammation is associated with secretion of TNF-α, which is linked with the development of pain [44]–[46]. The cytokine/chemokine profile of PapMV nanoparticles does not indicate any significant induction of TNF-α. This might suggest that injection of the adjuvant is painless. This is consistent with our observations; mice and ferrets immunized with PapMV nanoparticles did not show any signs of discomfort. PapMV nanoparticles can thus be viewed as a novel type of painless adjuvant inducing a balanced T_H1_/T_H2_ immune response. Because PapMV nanoparticles are produced in bacteria at a very high yield, we believe that this adjuvant has very promising potential because it can be produced in large quantities at a competitive cost.

The heterosubtypic protection that we observed could have been mediated through cross-reactive antibodies [12] and/or by influenza specific CD8+ T cells [11]. Several DNA vaccine studies have demonstrated that vaccination combining HA with DNA encoding internal proteins was more effective than immunization with individual plasmid alone [47]–[49]. A recent H5N1 DNA vaccine study in a mouse model showed that a combination of HA-antibody and a CD8+ CTL response directed against a specific NP epitope resulted in reduced inflammation severity and lung viral titers compared to mice in which only one arm of the immune system was activated [50]. However, DNA vaccines are not very efficient in large animals and need sophisticated electroporation technology to be used in humans, which is not compatible with broad vaccination campaign. The use of the PapMV nanoparticles is simple and does not require sophisticated equipment. PapMV nanoparticles need only to be added to the TIV before injection to benefit from an improved immune response. The effectiveness of the dose can be significantly augmented (4 fold), as well as the efficacy and the memory response of the vaccine toward heterosubtypic strains. In the case of a pandemic, it would be conceivable to use PapMV nanoparticles as an adjuvant to improve the protection afforded by the seasonal flu vaccine toward heterosubtypic strains to ensure a rapid protection of the population during the time needed to produce a new vaccine adapted to the pandemic strain. Once the pandemic vaccine is obtained, PapMV nanoparticles could continue to be used to decrease the doses of vaccine required and to augment coverage. To our knowledge, this is the first report describing the adjuvant property of plant-virus-derived nanoparticles.

## Materials and Methods

### Ethics Statement

All the work with animals has been done with Institution approved ethics protocol by the 'Comité de Protection des Animaux - CHUQ (CPA-CHUQ). The approval of this project is found under the authorization number 2010148-1.

### Production of PapMV nanoparticles

Expression and purification of PapMV nanoparticles were performed as described previously [17]. LPS contamination was always less than 5 endotoxin (EU) units/mg of protein. The size and structure of the nanoparticles were confirmed by observation on a TEM (JEOL -1010, Tokyo, Japan) and dynamic light scattering (DLS) (Zetasizer Nano ZS, Malvern, Worcestershire, UK) microscope.

### *In vivo* fluorescence imaging

Nanoparticles were labeled with Alexa@680 (Invitrogen, Burlington, ON, Canada) and injected (25 µg) into the footpad of 3 anesthetized Balb/C mice. Three other mice were injected with Alexa@680 staining as negative control. The images were gathered with an IVIS 200 imaging system (Xenogen, Alameda, CA, USA) at 24, 48 and 72 hours. The data are represented as pseudocolor images indicating fluorescence intensity (red and yellow, most intense).

### Cytokine/chemokine profile

Mice (Charles River, Wilmington, MA) were immunized (2 groups of 5 Balb/C mice) with 30 µg of PapMV nanoparticles once or twice at 2-week intervals. Splenocytes, 2.5 × 10^5^ cells/well were reactivated with either culture medium or nanoparticles and cultured for 36 h. The cytokines and chemokines were evaluated with MILLIPLEX MAP Mouse Cytokine/Chemokine - Premixed 22 Plex (Millipore, Billerica, MA, USA) for Luminex® xMAP® platform. Measurements were performed with a Luminex 100IS liquichip workstation (Qiagen, Canada).

### Immunization

Mice, five to ten Balb/C mice per group (Charles River, Wilmington, MA) were injected s.c. with 1/5 of the human dose of the seasonal (Fluviral; Glaxo Smith Kline) TIV (2007–2008; A/Solomon Islands/3/2006 (H1N1): A/Wisconsin/67/2005 (H3N2) and B/Malaysia/2506/2004), TIV (2008–2009; A/Brisbane/59/2007 (H1N1), A/Brisbane/10/2007 (H3N2), and B/Florida/4/2006) or TIV (2009–2010; A/Brisbane/59/2007 (H1N1), A/Brisbane/10/2007 (H3N2) and B/Brisbane/60/2008) alone or adjuvanted with 3 µg or 30 µg of PapMV nanoparticles. We also used the TIV (2008–2009) (Influvac) from Solvay. The animals were given two immunizations at 14–day intervals. Blood samples were collected at days 14 and 28. Ferrets were immunized twice at 3-week intervals with one human dose of TIV (2009–2010) alone or with 150 µg of PapMV nanoparticles by the intramuscular route. Blood was recovered 21 days after each immunization.

### Antibody titration by ELISA

ELISA was performed as previously described (11) using the following antigens: TIV at 0.1 µg/ml, GST-NP at 1 µg/ml, A/WSN/33 virions at 0.1 µg/ml, A/Kentucky/91(H3N8) virions (Flu Avert; Intervet) vaccine at 1 µg/ml. The GST-NP and GST-M1 antigens were produced through a C-terminal GST fusion with the NP and the M1 gene of the WSN/33 influenza strain and affinity purified.

### Immunoblot of WSN/33 HA overlapping peptides

A Nexterion® Slide AL from Microarray Solutions SCHOTT North America Inc. (Louisville, KY, USA) was used to spot 56 overlapping WSN/33 HA peptides (synthesized by GeneScript, Piscataway, NJ, USA) in duplicate. Slides were blocked with PBS + 0.05% Tween® 20 (PBST) + 1% BSA for 1 hr at RT. Testing (TIV + nanoparticles immunized ferrets) and control serums (TIV alone immunized ferrets) were added to the wells and incubated for 1 h30 at RT. Wells were washed three times for 3 minutes each with PBST. Alexa fluor 647 anti-IgG ferret at 20 µg/ml (anti-IgG ferret [Bethyl, cat#A140-108P] were added and incubated at RT for 1 hr, followed by four washes with PBST. The slides were dried and scanned with the apparatus ScanArray 4000XL (GSI Lumonics) and images were analyzed with GenePix Pro. Ratio F555 mean normalized  =  mean (signal intensity of the testing serum– background around the dot)/normalization signal mean (signal intensity of the control serum - background around the dot). A ratio F555 ≥3 was considered a positive signal.

### Splenocyte isolation and ELISPOT assays

The day before splenocyte isolation, ethanol (70%)-treated MultiScreen-IP opaque 96-well plates (High Protein Binding Immobilon-P membrane, Millipore, Bedford, MA) were coated overnight at 4°C with 100 µl/well of capture IFN-γ antibody, diluted in DPBS (Abcam, Cambridge, MA, USA). Two weeks after the boost, mouse spleen cells were isolated. The precursor frequency specific T cells was determined by subtracting the background spots in media alone from the number of spots seen in response to the different activators.

### Influenza A and challenge

The influenza virus A strain used in this study is A/WSN/33 (H1N1). Mice were infected by the intranasal route using 50 µl containing 1LD50. Mice were monitored daily for clinical symptoms (loss of body weight, abnormal behavior and ruffled fur). Deaths were recorded over a period of 14 days.

### CD8+ T-cell depletion

For T-cell depletion, we injected mice with 0.1 mg i.p. of monoclonal antibodies directed to CD8+ in vaccinated mice at day 33 and 35. After depletion, validated by FACS, mice were challenged as before on day 36.

### Statistical analysis

Data were analyzed with parametric (or non parametric when the variance were significantly different) ANOVA test. Tukey's post tests were used to compare differences (antibody titers, ELISPOT) among groups of mice. Differences among survival curves were analyzed by Kaplan–Meier survival analysis. Values of *p < 0.05, **p < 0.01, ***p < 0.0001 were considered statistically significant. Statistical analyses were performed with GraphPad PRISM 5.01.

## Supporting Information

Figure S1 **Purification of the PapMV CP from bacteria.** **A,** SDS-PAGE purification of PapMV CP over-expressed in *E. coli*. Lanes: 1 Broad range protein marker, 2 bacterial lysate before induction, 3 bacterial lysate after induction, 4 purified PapMV CP after elution **B,** Size distribution of PapMV nanoparticles as measured by dynamic light scattering (DLS) showing a peak at 70 nm.
(TIF)

Figure S2 **SDS-PAGE purification profile of GST-NP.** The NP gene of the influenza strain WSN/33 was cloned in fusion with the C-terminus of GST. GST-NP was expressed in *E. coli*. GST-NP was used to evaluate the antibody titer to NP by ELISA and IFN-γ secretion by ELISPOT. Lanes: 1 Broad range protein marker, 2 bacterial lysate before induction, 3 bacterial lysate after induction, 4 purified GST-NP after elution.
(TIF)

Figure S3 **Alum as an adjuvant of TIV (2007–2008).** Balb/C mice (10 per group) were vaccinated once (s.c.) with 1/5 of the human dose of the TIV (2007–2008) or adjuvanted alum. Serum was collected 14 days after immunization. Total IgG (**A**) or the IgG2a subtype (**B**) were measured by ELISA against TIV (2007–2008).
(TIF)

Figure S4 **PapMV nanoparticles improve the humoral response of TIV (2008–2009).** Balb/C mice (10 per group) were vaccinated twice with a 14-day interval with 1/5 of the human dose of TIV (2008–2009) adjuvanted with 30 µg PapMV nanoparticles. Serum collected 14 days after the boost was analyzed by ELISA, measuring total IgG titers (**A**) and IgG2a titers (**B**) directed towards TIV (2008–2009). IgG2a titers directed towards purified recombinant GST-NP [A/WSN/33 (H1N1)] were also measured (**C**). * p< 0.05,** p< 0.01, *** p< 0.001. Numbers (>3X, >4X, >16X) represent the fold increase of antibodies in the adjuvanted group as compared to TIV (2008–2009) alone.
(TIF)

Figure S5 **PapMV nanoparticles improve the humoral response of TIV (2009–2010).** Balb/C mice (5 per group) were vaccinated twice with a 14-day interval with 1/5 of the human dose of TIV (2009–2010) adjuvanted with 30 µg PapMV nanoparticles. The humoral response was analysed by ELISA using serum collected 14 days after the boost. We measured total IgG titers (**A**) and IgG2a titers (**B**) directed towards TIV (2009–2010). IgG2a titers directed towards purified recombinant GST-NP (A/WSN/33 (H1N1)) were also measured (**C**) as well as total IgG titers directed towards the pandemic influenza vaccine 2009 (**D**). *** p < 0.001. Numbers represent the fold increase of antibodies in the adjuvanted group as compared to TIV (2009–2010) alone.
(TIF)

Figure S6 **Immunoblot analysis using 56 peptides** (15-mers overlapping by 5 amino acids and derived from the HA of the influenza strain WSN/33) exposed to serum of ferrets immunized with TIV (2009–2010) alone (**A**) or TIV (2009–2010) adjuvanted with 150 µg of PapMV nanoparticles (**B**). The binding of IgG was revealed with an anti-ferret antibody conjugated to a fluorescent dye. The fluorescence is showed in black and white in panels **A** and **B**. Panel **C** shows an overlay of fluorescence obtained with TIV (2009–2010) treatment stained in red and fluorescence obtained with the adjuvanted group strained in green in order to visualize the signals with a better contrast.
(TIF)

Figure S7 **Challenge of vaccinated mice with the heterosubtypic strain A(H1N1) WSN/33.** Mice (10 per group) were vaccinated twice with 1/5 of the human dose of commercial TIV (2008–2009) from 2 different companies (#1 and # 2) with or without 30 µg of PapMV nanoparticles. Mice were challenged with 1LD50 of A(H1N1)/WSN/33 influenza virus 2 weeks after the last boost and were followed for a 14-day period. **A,** Body weight of mice, expressed as percentage of initial weight. **B,** Symptoms observed on each infected mouse were scored each day after the challenge. Symptoms: 0. No symptoms. 1. Lightly spiked fur, slightly curved back. 2. Spiked fur, curved back. 3 Spiked fur, curved back, difficulty in moving and mild dehydration. 4. Spiked fur, curved back, difficulty in moving, severe dehydration, closed eyes and ocular secretion.
(TIF)

Figure S8 Long lasting humoral response in mice. Mice (10 per group) were vaccinated once with 1/5 of the human dose of commercial TIV (2007–2008) with or without 30 µg of PapMV nanoparticles. The results presented here refer to the humoral response 10 months after immunization. **A,** Total IgG directed to TIV (2007–2008). **B,** IgG2a titer directed to TIV (2007–2008), and **C,** IgG2a titer directed to WSN/33 GST-NP antigen. Mice were challenged with 1LD50 of (H1N1) WSN/33 influenza virus, 10 months after the last immunization and were analyzed for a 14-day period. **D**) Body weight of mice, expressed as percentage of initial weight. **E**) Symptoms (defined in legend to Figure S6) observed on each infected mouse were scored each day after the challenge.
(TIF)

Figure S9 PapMV nanoparticles induce a CTL response to conserved influenza proteins when used as an adjuvant in TIV (2009–2010). Mice (10 per group) were vaccinated twice with 1/5 of the human dose of commercial TIV (2009–2010) with or without 30 µg of PapMV nanoparticles. To verify the importance of the CTL response, we depleted CD8+ cells from vaccinated mice by injecting 0.1 mg of anti-CD8+ antibody. Mice were challenged with 1LD50 of A(H1N1)/WSN/33 influenza virus 2 weeks after the last immunization and were analyzed for a 14-day period. **A,** Body weight of mice, expressed as a percentage of initial weight. **B,** Symptoms (defined in legend to Figure S6) observed on each infected mouse were scored daily after the challenge. **C,** Correlation analysis of IgG titer against WSN/33 (H1N1) as a function of highest body weight loss (%) during the challenge.
(TIF)
