# Supplementary figures and images for: Improvement of the Trivalent Inactivated Flu Vaccine Using PapMV Nanoparticles
Source: PLoS One. 2011 Jun 29;6(6):e21522. doi: 10.1371/journal.pone.0021522 (PMC3126827; doi:10.1371/journal.pone.0021522)

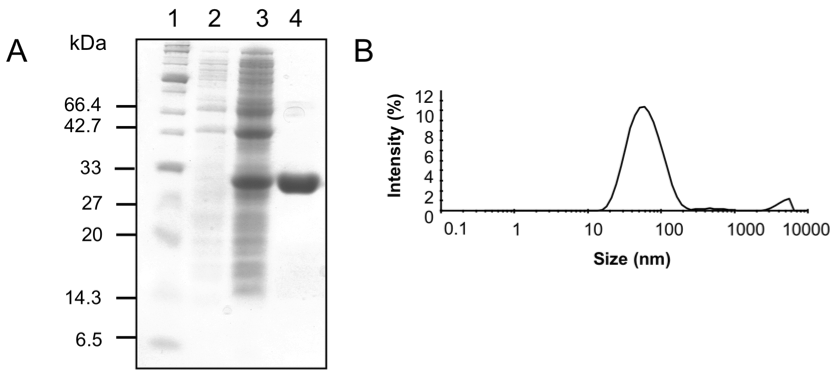

Supplement: Figure S1 — Purification of the PapMV CP from bacteria. A, SDS-PAGE purification of PapMV CP over-expressed in E. coli. Lanes: 1 Broad range protein marker, 2 bacterial lysate before induction, 3 bacterial lysate after induction, 4 purified PapMV CP after elution B, Size distribution of PapMV nanoparticles as measured by dynamic light scattering (DLS) showing a peak at 70 nm. (TIF) [file pone.0021522.s001.tif]

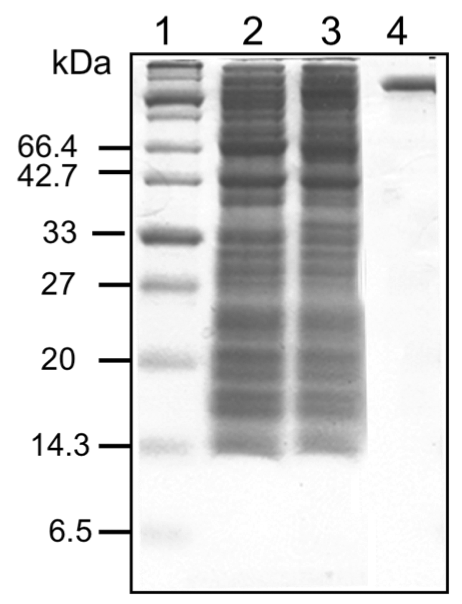

Supplement: Figure S2 — SDS-PAGE purification profile of GST-NP. The NP gene of the influenza strain WSN/33 was cloned in fusion with the C-terminus of GST. GST-NP was expressed in E. coli. GST-NP was used to evaluate the antibody titer to NP by ELISA and IFN-γ secretion by ELISPOT. Lanes: 1 Broad range protein marker, 2 bacterial lysate before induction, 3 bacterial lysate after induction, 4 purified GST-NP after elution. (TIF) [file pone.0021522.s002.tif]

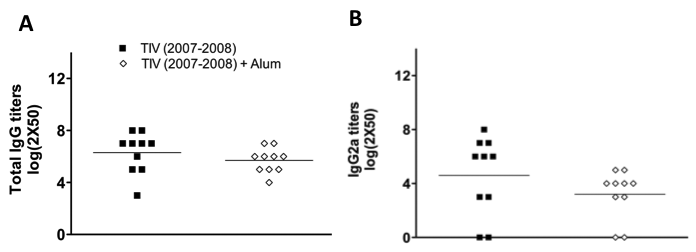

Supplement: Figure S3 — Alum as an adjuvant of TIV (2007–2008). Balb/C mice (10 per group) were vaccinated once (s.c.) with 1/5 of the human dose of the TIV (2007–2008) or adjuvanted alum. Serum was collected 14 days after immunization. Total IgG (A) or the IgG2a subtype (B) were measured by ELISA against TIV (2007–2008). (TIF) [file pone.0021522.s003.tif]

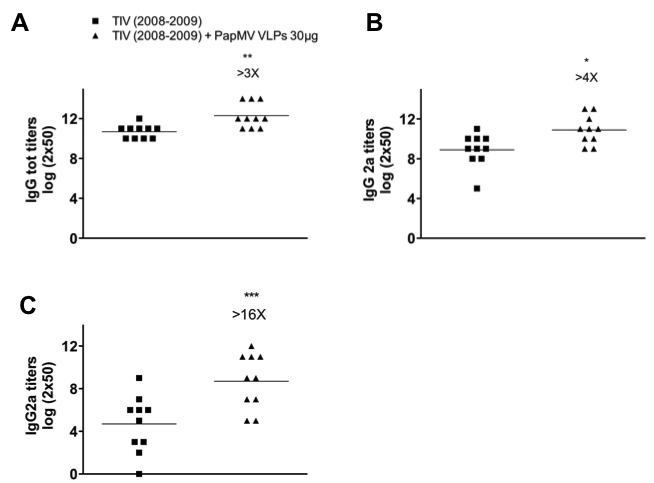

Supplement: Figure S4 — PapMV nanoparticles improve the humoral response of TIV (2008–2009). Balb/C mice (10 per group) were vaccinated twice with a 14-day interval with 1/5 of the human dose of TIV (2008–2009) adjuvanted with 30 µg PapMV nanoparticles. Serum collected 14 days after the boost was analyzed by ELISA, measuring total IgG titers (A) and IgG2a titers (B) directed towards TIV (2008–2009). IgG2a titers directed towards purified recombinant GST-NP [A/WSN/33 (H1N1)] were also measured (C). * p< 0.05,** p< 0.01, *** p< 0.001. Numbers (>3X, >4X, >16X) represent the fold increase of antibodies in the adjuvanted group as compared to TIV (2008–2009) alone. (TIF) [file pone.0021522.s004.tif]

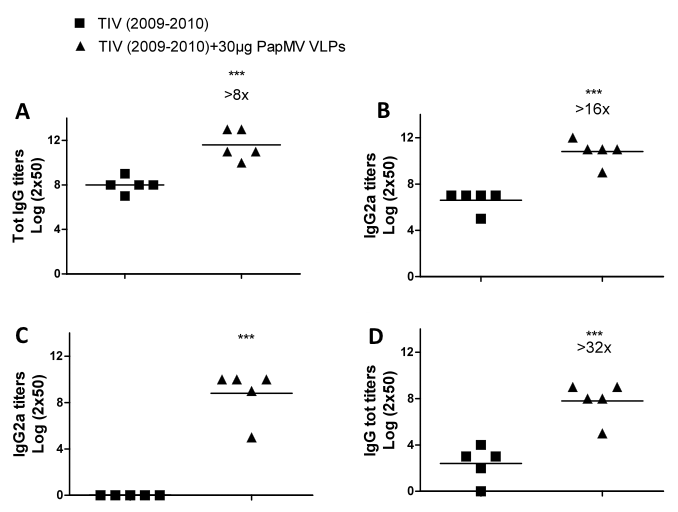

Supplement: Figure S5 — PapMV nanoparticles improve the humoral response of TIV (2009–2010). Balb/C mice (5 per group) were vaccinated twice with a 14-day interval with 1/5 of the human dose of TIV (2009–2010) adjuvanted with 30 µg PapMV nanoparticles. The humoral response was analysed by ELISA using serum collected 14 days after the boost. We measured total IgG titers (A) and IgG2a titers (B) directed towards TIV (2009–2010). IgG2a titers directed towards purified recombinant GST-NP (A/WSN/33 (H1N1)) were also measured (C) as well as total IgG titers directed towards the pandemic influenza vaccine 2009 (D). *** p < 0.001. Numbers represent the fold increase of antibodies in the adjuvanted group as compared to TIV (2009–2010) alone. (TIF) [file pone.0021522.s005.tif]

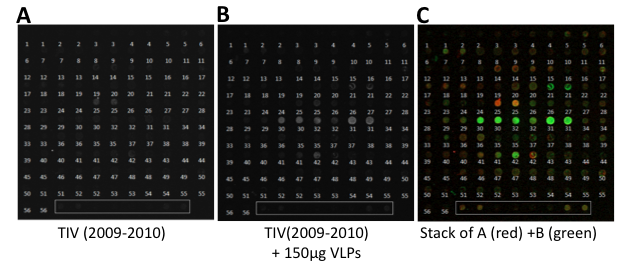

Supplement: Figure S6 — Immunoblot analysis using 56 peptides (15-mers overlapping by 5 amino acids and derived from the HA of the influenza strain WSN/33) exposed to serum of ferrets immunized with TIV (2009–2010) alone (A) or TIV (2009–2010) adjuvanted with 150 µg of PapMV nanoparticles (B). The binding of IgG was revealed with an anti-ferret antibody conjugated to a fluorescent dye. The fluorescence is showed in black and white in panels A and B. Panel C shows an overlay of fluorescence obtained with TIV (2009–2010) treatment stained in red and fluorescence obtained with the adjuvanted group strained in green in order to visualize the signals with a better contrast. (TIF) [file pone.0021522.s006.tif]

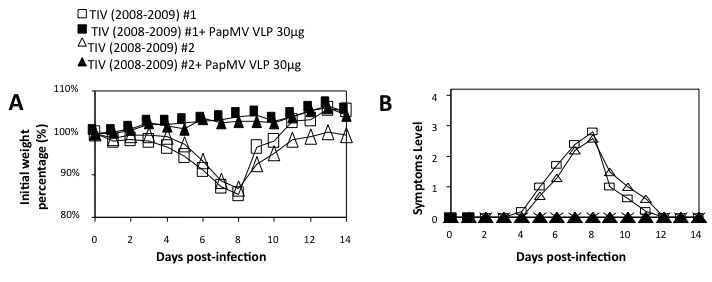

Supplement: Figure S7 — Challenge of vaccinated mice with the heterosubtypic strain A(H1N1) WSN/33. Mice (10 per group) were vaccinated twice with 1/5 of the human dose of commercial TIV (2008–2009) from 2 different companies (#1 and # 2) with or without 30 µg of PapMV nanoparticles. Mice were challenged with 1LD50 of A(H1N1)/WSN/33 influenza virus 2 weeks after the last boost and were followed for a 14-day period. A, Body weight of mice, expressed as percentage of initial weight. B, Symptoms observed on each infected mouse were scored each day after the challenge. Symptoms: 0. No symptoms. 1. Lightly spiked fur, slightly curved back. 2. Spiked fur, curved back. 3 Spiked fur, curved back, difficulty in moving and mild dehydration. 4. Spiked fur, curved back, difficulty in moving, severe dehydration, closed eyes and ocular secretion. (TIF) [file pone.0021522.s007.tif]

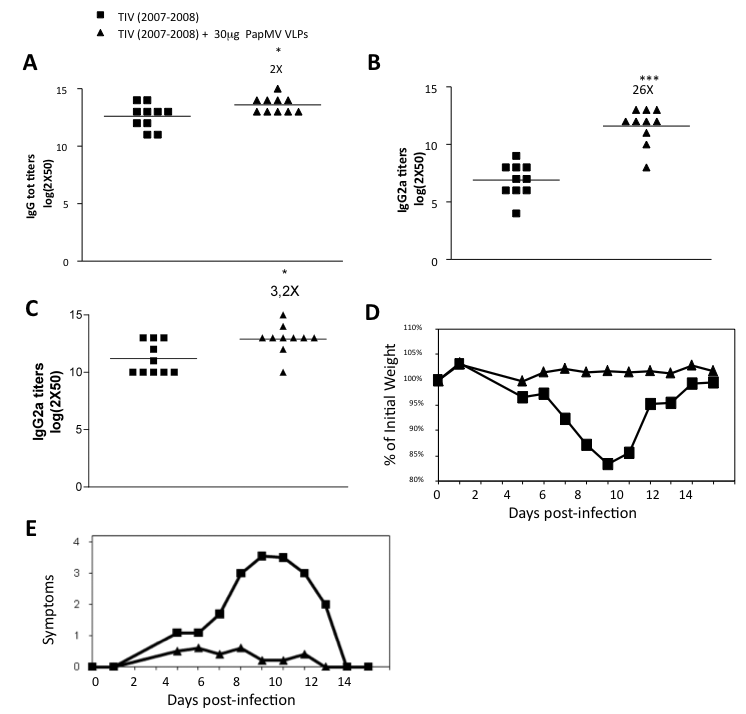

Supplement: Figure S8 — Long lasting humoral response in mice. Mice (10 per group) were vaccinated once with 1/5 of the human dose of commercial TIV (2007–2008) with or without 30 µg of PapMV nanoparticles. The results presented here refer to the humoral response 10 months after immunization. A, Total IgG directed to TIV (2007–2008). B, IgG2a titer directed to TIV (2007–2008), and C, IgG2a titer directed to WSN/33 GST-NP antigen. Mice were challenged with 1LD50 of (H1N1) WSN/33 influenza virus, 10 months after the last immunization and were analyzed for a 14-day period. D) Body weight of mice, expressed as percentage of initial weight. E) Symptoms (defined in legend to Figure S6) observed on each infected mouse were scored each day after the challenge. (TIF) [file pone.0021522.s008.tif]

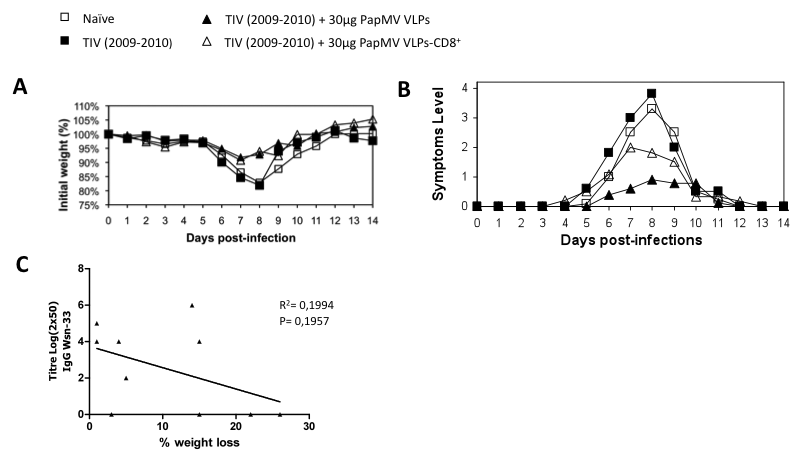

Supplement: Figure S9 — PapMV nanoparticles induce a CTL response to conserved influenza proteins when used as an adjuvant in TIV (2009–2010). Mice (10 per group) were vaccinated twice with 1/5 of the human dose of commercial TIV (2009–2010) with or without 30 µg of PapMV nanoparticles. To verify the importance of the CTL response, we depleted CD8+ cells from vaccinated mice by injecting 0.1 mg of anti-CD8+ antibody. Mice were challenged with 1LD50 of A(H1N1)/WSN/33 influenza virus 2 weeks after the last immunization and were analyzed for a 14-day period. A, Body weight of mice, expressed as a percentage of initial weight. B, Symptoms (defined in legend to Figure S6) observed on each infected mouse were scored daily after the challenge. C, Correlation analysis of IgG titer against WSN/33 (H1N1) as a function of highest body weight loss (%) during the challenge. (TIF) [file pone.0021522.s009.tif]
